# Geographic remoteness, area-level socioeconomic disadvantage and inequalities in colorectal cancer survival in Queensland: a multilevel analysis

**DOI:** 10.1186/1471-2407-13-493

**Published:** 2013-10-24

**Authors:** Peter D Baade, Paramita Dasgupta, Joanne F Aitken, Gavin Turrell

**Affiliations:** 1 Cancer Council Queensland, Brisbane, Australia; 2 School of Public Health and Social Work, Queensland University of Technology, Brisbane, Australia; 3 Griffith Health Institute, Griffith University, Gold Coast, Australia; 4 School of Population Health, University of Queensland, Brisbane, Australia; 5 Senior Research Fellow, Cancer Council Queensland, PO Box 201, Spring Hill, QLD 4001, Australia

**Keywords:** Colorectal cancer, Epidemiology, Survival, Inequalities, Multilevel

## Abstract

**Background:**

To explore the impact of geographical remoteness and area-level socioeconomic disadvantage on colorectal cancer (CRC) survival.

**Methods:**

Multilevel logistic regression and Markov chain Monte Carlo simulations were used to analyze geographical variations in five-year all-cause and CRC-specific survival across 478 regions in Queensland Australia for 22,727 CRC cases aged 20–84 years diagnosed from 1997–2007.

**Results:**

Area-level disadvantage and geographic remoteness were independently associated with CRC survival. After full multivariate adjustment (both levels), patients from remote (odds Ratio [OR]: 1.24, 95%CrI: 1.07-1.42) and more disadvantaged quintiles (OR = 1.12, 1.15, 1.20, 1.23 for Quintiles 4, 3, 2 and 1 respectively) had lower CRC-specific survival than major cities and least disadvantaged areas. Similar associations were found for all-cause survival. Area disadvantage accounted for a substantial amount of the all-cause variation between areas.

**Conclusions:**

We have demonstrated that the area-level inequalities in survival of colorectal cancer patients cannot be explained by the measured individual-level characteristics of the patients or their cancer and remain after adjusting for cancer stage. Further research is urgently needed to clarify the factors that underlie the survival differences, including the importance of geographical differences in clinical management of CRC.

## Background

Worldwide, colorectal cancer (CRC) was the second most common invasive cancer in 2008 and the fourth most deadly form of cancer [1]. Advances in cancer prevention, screening, and management over recent decades [2] have contributed to the ongoing improvements in CRC survival in developed countries [1] with Australia having one of the highest survival rates globally [3]. However not all patients have benefited equally from these advances, with international studies consistently reporting survival inequalities by area disadvantage and heath care access, [4-6] with evidence that these inequalities may be widening [7]. Australians living outside major cities, in socioeconomically disadvantaged regions or further away from radiation facilities also have poorer survival after a diagnosis of colon or rectal cancer [8-11]. Inequities in oncology services and general health care provision with increasing geographical isolation in Australia have been well documented [12] and acknowledged to be contributing factors to the greater burden for remote cancer patients.

Nonetheless, relatively few studies have quantified the impact that area-level factors have on geographical inequalities in CRC survival while specifically considering the effect of the underlying nested geographical structure [5,13]. Multilevel models enable us to simultaneously estimate the impact of both individual- and area-level explanatory variables on the total variation in individual outcomes while accounting for the clustering of observations within the same geographical location. Improved computing capacity has led to the increasing adoption of sophisticated multilevel techniques for large-scale population-based studies to quantify geographical inequalities in cancer survival and explore underlying causes [5,13-15].

A recent study examined the extent of spatial variation in CRC relative survival across small areas in Queensland [16]. However, that study was designed primarily to measure the impact that spatial variations had on premature mortality and so utilized data aggregated over each region and combinations of covariates. This removed any opportunity to simultaneously examine the impact that area- and individual-level factors had on differences in survival between individual patients.

In this study we explore whether geographical remoteness and socioeconomic characteristics of the area where a CRC patient resides at diagnosis are associated with their survival, independently of the characteristics of the individual patients themselves. To the best of our knowledge it is the first Australian study to quantitatively assess the independent associations between the characteristics of geographical areas and the characteristics of individuals in those areas with survival.

Specifically we aimed to:

i. assess whether all-cause and CRC-specific survival varied with a patient’s area of residence while controlling for within-area variation in individual effects and between-group variation in area-level factors;

ii. explore the independent impact of remoteness and area disadvantage on survival after adjusting for individual characteristics;

iii. identify individual-level factors influencing CRC survival; and

iv. explore the effect of interactions between area-level factors on survival.

Being able to quantify geographical variations in survival and identify associations with these disparities may assist advocates and health planners to develop strategic plans and public health interventions to reduce these inequalities.

## Methods

Ethical approval to conduct this study was obtained from the University of Queensland Social and Behavioral Sciences Ethical Review Committee. Queensland Health provided legislative approval to access routinely collected population-based cancer data in Queensland.

### Study cohort

All incident cases of invasive CRC (ICD-O3 codes C18 to C20, C218) diagnosed between January 1, 1997 and December 31, 2007 (inclusive) were extracted from the state-wide population-based Queensland Cancer Registry to which all confirmed invasive cancers diagnosed among Queensland residents must be legally reported. Data quality is high as evidenced by the low percentage (1.4%) of death certificate notifications only and high percentage (92.1%) of histologically verified cases in 2007 [17]. We restricted our cohort to those aged between 20 and 84 years at diagnosis since CRC is relatively rare among younger age groups, while among older patients death certificates are less accurate [18] and their clinical management is different [19,20]. Cases were excluded if they were notified by death certificate only, were first identified at autopsy or could not be geocoded. For patients with multiple primary colon or rectal cancers, only the tumor with the most advanced stage was considered. Variables extracted (categories in Table 1) included year and age at diagnosis, gender, occupation, marital status, country of birth, CRC site (colon C18; rectum C19-C20,C218), differentiation and Indigenous status, with the latter being considered sufficiently complete for analysis [21].

**Table 1:** Cohort description and unadjusted five year estimates of all-cause and colorectal cancer-specific outcomes for colorectal cancer patients aged 20–84 in Queensland, 1997–2007

		**All-cause**			**Colorectal cancer**		
sub group	N (%)	deaths (%)	survival [95% CI]	p	deaths (%)	survival [95% CI]	p
All patients in cohort	22,727	41.1	58.1 [57, 58]		31.8	66.3 [66, 67]	
***Area-Remoteness Index of Australia (ARIA)***				*< 0.001*			*< 0.001*
Major city	13,155 (57.9)	39.6	59.6 [59, 60]		30.0	68.1 [68, 69]	
Inner regional	5,139 (22.6)	41.4	57.8 [56, 59]		32.2	65.9 [65, 67]	
Outer regional	3,485 (15.3)	45.1	54.1 [52,56]		36.0	61.6 [60, 63]	
Remote^1^	948 (4.2)	46.2	53.1 [50,56]		38.2	59.6 [56, 63]	
***Index of Relative socioeconomic advantage and disadvantage (IRSAD)***				*< 0.001*			*< 0.001*
Quintile 5 (least disadvantaged)	3,193 (14.1)	36.4	62.8 [61, 65]		28.0	70.4 [69, 72]	
Quintile 4	5,101 (22.4)	38.9	60.2 [59, 62]		29.8	68.3 [67, 70]	
Quintile 3	6,075 (26.7)	41.0	58.2 [57, 59]		32.2	65.9 [65, 67]	
Quintile 2	5,335 (23.5)	44.5	54.6 [53,56]		34.5	63.3 [62, 65]	
Quintile 1 (most disadvantaged)	3,023 (13.3)	43.8	55.4 [54,57]		33.5	64.2 [62, 66]	
***Age group***				*< 0.001*			*< 0.001*
20 to 49	1,873 (8.2)	32.0	67.4 [65, 70]		29.3	69.8 [68, 72]	
50 to 59	3,938 (17.3)	32.8	66.7 [65, 68]		29.7	69.3 [68, 71]	
60 to 69	6,578 (28.9)	37.1	62.1 [61, 63]		30.7	67.8 [67, 69]	
70 to79	7,718 (34.1)	45.6	53.5 [52,55]		32.4	64.8 [64, 66]	
80 to 84	2,620 (11.5)	56.7	41.9 [40,44]		37.7	58.4 [56, 60]	
***Gender***				*< 0.001*			*=0.003*
Male	12,879 (56.7)	42.9	56.2 [55,57]		32.5	65.1 [64, 66]	
Female	9,848 (43.3)	38.8	60.6 [60, 62]		30.8	67.7 [67, 69]	
**I*ndigenous status***				*< 0.001*			*< 0.001*
Non Indigenous	20,868 (91.8)	43.1	56.1 [55,57]		33.4	64.5 [64, 65]	
Indigenous	181 (0.8)	45.3	53.7 [45, 61]		35.4	63.1 [55, 70]	
Not stated	1,678 (7.4)	16.7	82.9 [81, 85]		11.3	88.3 [87, 90]	
***Marital status***				*<0.001*			*< 0.001*
Married	14,532 (63.9)	39.0	60.1 [59, 61]		30.8	67.4 [67, 68]	
Never married/single	1,541 (6.8)	46.5	52.6 [50,55]		36.2	61.5 [59, 64]	
Widowed	3,951 (17.4)	48.2	51.1 [49,52]		34.2	63.2 [62, 65]	
Divorced	1,822 (8)	44.4	54.7 [52,57]		36.2	61.6 [59, 64]	
Separated	454 (2)	31.9	67.3 [63, 71]		24.4	74.3 [70, 78]	
Not stated	427 (1.9)	20.6	79.3 [75, 83]		15.5	84.2 [80, 87]	
***Occupation category***				*< 0.001*			*< 0.001*
Professional	4,783 (21.1)	48.6	50.6 [49,52]		39.0	58.4 [57, 60]	
White collar	2,665 (11.7)	52.6	46.7 [44,49]		41.4	55.6 [54, 58]	
Blue Collar	3,789 (16.7)	59.5	39.4 [38,41]		47.0	48.9 [47,51]	
Not in labor force	7,529 (33.1)	33.5	65.9 [65, 67]		25.5	73.3 [72, 74]	
Not stated/ Inadequately described	3,961 (17.4)	21.0	78.2 [77, 79]		14.0	85.2 [84, 86]	
***Country birth***^***2***^				*< 0.001*			*< 0.001*
Australia	17,367 (76.4)	41.9	57.2 [57, 58]		32.3	65.7 [65, 66]	
Other English-speaking	4,580 (20.2)	39.2	60.2 [59, 62]		30.7	67.8 [66, 69]	
Non-English-speaking	780 (3.4)	34.0	64.2 [61, 68]		26.4	71.1 [67, 74]	
***Site***^***3***^				*=0.003*			*< 0.001*
Proximal(R) colon	7,874 (34.6)	41.8	57.5 [56, 59]		31.7	66.4 [64, 67]	
Distal (L) colon	5,865 (25.9)	39.5	59.6 [58, 61]		30.0	68.8 [68,70]	
Colon NOS	1,299 (5.7)	54.0	45.3 [43,48]		44.5	53.2 [50,56]	
Rectal	7,689 (33.8)	39.4	59.8 [59, 60]		31.0	66.9 [65, 68]	
***Stage***				*< 0.001*			*< 0.001*
Stage A	4,332 (19.1)	18.3	81.1 [80, 83]		7.8	91.7 [91, 93]	
Stage B	6,323 (27.8)	28.9	70.3 [69, 71]		18.1	80.4 [79, 81]	
Stage C	5,846 (25.7)	47.9	50.8 [50,52]		39.6	57.5 [56, 58]	
Stage D	2,576 (11.3)	84.7	13.9 [12,15]		80.6	15.6 [14,17]	
Unknown stage	3,650 (16.1)	47.4	51.9 [50,54]		36.9	60.9 [59, 62]	
***Differentiation***				*< 0.001*			*< 0.001*
Well differentiated	1,107 (4.9)	31.9	67.3 [65, 70]		20.1	75.9 [76, 81]	
Moderate differentiated	13,953 (61.4)	36.7	62.4 [62, 63]		27.4	70.7 [70, 71]	
Poor differentiated	4,206 (18.5)	52.9	46.2 [45,48]		44.2	53.3 [52,55]	
Not stated	3,461 (15.2)	47.2	52.2 [50,54]		38.0	60.3 [59, 62]	
***Surgical margins***				*< 0.001*			*< 0.001*
Clear	16,664 (73.4)	36.3	62.9 [62, 64]		26.9	71.2 [70, 72]	
Positive	530 (2.3)	39.8	59.7 [55, 61]		31.1	67.3 [63, 71]	
Unknown	5,533 (24.3)	55.7	43.6 [43,45]		46.4	51.2 [50,53]	
***Distance to Treatment***				*< 0.001*			*< 0.001*
0-99 km	16,042 (70.5)	39.4	59.7 [59, 61]		30.1	68.1 [67, 69]	
100-399 km	5,173 (22.8)	44.9	54.4 [53,56]		35.2	62.3 [61, 64]	
400 or more km	1,512 (6.7)	45.7	53.5 [51,54]		37.8	60.3 [58, 62]	

CI = confidence interval; p-values calculated using log-rank test for equality of survivor functions restricting follow-up to five years for each patient.

^1^ Includes remote and very remote categories.

^2^ Other English-speaking: those born in New Zealand, United Kingdom, Ireland, or North America; non-English-speaking: those not born in Australia, New Zealand, United Kingdom, Ireland or North America.

^3^ Colorectal sites defined as: proximal colon (ICDO3: C180 to C184), distal colon (ICDO3: C185-C187), unspecified colon (ICDO3: C188-C189) and rectal (ICDO3: C19-C20, C218).

### Geocoding and travel distance calculations

Residential addresses were geocoded using full street address (98.0% of cases), a street at the center of the suburb (1.8%) or the post code (0.2%) at diagnosis. Radiotherapy facilities in Queensland are concentrated in larger cities and typically affiliated to major cancer care centers; hence these distances are a proxy measure of access to optimum cancer treatment. Geographical Information System software and a street network database were used to calculate road travel distances from each patient’s geocoded location to the closest radiotherapy facility as described previously [8]. These road travel distances represent the minimum distance, since it is possible that some patients may not have chosen the closest facility for treatment.

### Survival data

The study cohort was followed up to 31st December 2010. The Queensland Cancer Registry routinely all incident cases to the Registrar of Births, Deaths and Marriages and the National Death Index to ascertain mortality status for all cancer patients diagnosed in Queensland [17]. Additional data from hospitals and pathology records are used to finalize the cause of death thereby providing a high degree of accuracy; although as with all population-based registries cause of death misclassification remains a possibility. Survival was measured in years from date of diagnosis to death or the study end point. Deaths from other causes were censored when estimating CRC-specific survival. The follow up time for patients who survived more than five years after diagnosis was censored at five years.

### Extraction of clinical data from pathology forms

#### Stage at diagnosis

As has been previously described, [8] information extracted from pathology forms [22] was used to categorize stage at diagnosis into four groups ranging from Stage I (least advanced) to IV (metastatic) based on the TNM system [23].

#### Surgical margins

The recorded information on the distance between the tumor and outer edge of tissue sample removed during biopsy or CRC resection was used to categorize patients as having clear (no cancer cells at outer edge of sample), positive (cancer cells present at or close to the edge) or unknown surgical margins. Cancers that are recorded with a clear margin are deemed to have been completely excised which has been shown to be associated with lower recurrence and better survival [24].

### Geographical area

Statistical Local Areas (SLA, n = 478) were used as the geographical unit for this study as they are deemed to be relatively homogenous with respect to population characteristics and socioeconomic status. In 2006 there were 478 SLAs in Queensland with a median population of 5,810. Cancer incidence data across all years were mapped to the 2006 SLA boundaries based on geocoded location at diagnosis thereby removing any impact of temporal changes in geographic boundaries.

#### Area-level socioeconomic disadvantage

Each patient was assigned to a quintile of area disadvantage (of increasing advantage from Quintile 1) based on the Australian Bureau of Statistics-derived Index of Relative Socioeconomic Advantage and Disadvantage (IRSAD). The IRSAD is determined from census measures related to both advantage and disadvantage, such as the proportion of tertiary educated residents and the proportion of low income households in a SLA [25]. This index was chosen as it does not include Indigenous status in its derivation.

#### Geographic remoteness

The address of usual residence at CRC diagnosis was grouped according to their level of geographic remoteness using the Australian Standard Geographical Classification Remoteness Index [26], which is a purely geographic measure of remoteness based on road distances from population centers to various levels of service provision (see Table 1 for categories).

### Statistical analysis

The five-year all-cause and CRC-specific survival rates were assessed using Kaplan-Meir analysis and estimates compared across patient-sub groups with the log rank test.

### Discrete-time multilevel logistic survival models

We carried out a full discrete-time multilevel logistic survival analysis that retained the underlying nested structure. This approach differs markedly from frailty models [27] where random effects are used to model clustering effects by adjusting the standard errors to account for non-independence of data within clusters. Frailty models offer a more restricted approach than multilevel methods [28,29]. Multilevel models use measures of clustering and variance in informative ways and can simultaneously model and partition the observed variation across individual and area-levels [28,30,31]. They are increasingly the method of choice when analysing data with a clear hierarchical structure [32].

Discrete-time multilevel logistic survival models are fitted to an expanded person-period dataset, containing a sequence of binary responses for each individual from each event time (in years). This variable is coded as 1 if an individual dies during a time interval t (measured in years in current analysis) and zero otherwise. Therefore a patient who is censored is indicated by a sequence of zeros for each t while one who dies will be denoted by value 1 for year in which death occurred and zero for each previous year. Once an individual dies data collection stops for that person. Hence the discrete response for a person who died during the third year following diagnosis would be (0, 0, 1), whereas it would be (0, 0, 0) if an individual was censored that year. Discrete-time multilevel survival models are thus equivalent to fitting a logistic regression model to the expanded dataset [14,33]. The restriction of follow-up to 5 years enabled the efficient computation of these complex models, which can be problematic when using this approach for analyzing large population-based datasets with long follow up intervals [33].

Although continuous time models remain the most popular for survival analysis, discrete-time methods have several advantages, especially in the multilevel framework when using large public health data sets [33,34]. Generating an expanded person-time dataset using months or days, instead of years, would increase the size of the dataset by more than ten or 300-fold [34]. Given the size of our initial data set, this additional expansion was not feasible. It is for this reason that discrete-time methods, using years as the time variable, are preferred in the multilevel framework.

The hazard function for a discrete-time multilevel logistic survival model is the conditional probability of death in interval t given that no death has occurred in the previous intervals [33,34]. When the hazard is modelled using the logit link, the exponentiated regression parameters are interpreted as the odds ratios (OR) rather than hazard ratios. Although the baseline hazard can be modelled using dummy variables for each time interval, in practice efficient estimates of model parameters can be obtained using low order polynomials for the time [35].

When the hazard is small, which is often the case if time intervals are narrow or probability of death occurring in time interval t is low (i.e. death is a rare event), the parameter estimates from the logit and Cox models are likely to comparable. Hence discrete-time logistic regression may be considered as an approximation to the Cox model [33,34]. An additional table demonstrates this. (see Additional file 1, Additional file 2).

Discrete-time multilevel logistic survival analysis was used to quantify the effect of area disadvantage and geographic remoteness on all-cause and CRC-specific survival after adjusting for individual-level factors. Models were fitted using Markov chain Monte Carlo (MCMC) [36] simulations in MLwiN version 2.26 [37] (University of Bristol, United Kingdom) interfaced with Stata (StataCorp, Texas) [38]. Convergence was assessed by trace and density plots, the autocorrelation of model parameters from posterior distributions and diagnostic tests [36] with none indicating non-convergence. After a burn in period of 40,000 iterations, parameter estimates were obtained from a further 80,000 iterations (with every 10th iteration kept). The underlying hazard was described with a second-order polynomial (i.e. time (years) and time-squared) [14].

A systematic three-step approach was used for each outcome. First we estimated null models that comprised individuals nested in SLAs without covariates. A significant area-level random term (based on the Wald χ^2^) [37] suggested that the modeled survival rates vary across areas in Queensland. We then added individual covariates before including area-level remoteness and neighborhood disadvantage (separately or simultaneously) with the full model being simultaneously adjusted for all explanatory variables on both levels. Interactions were tested (Wald χ^2^) by including both second-order terms and main-effects of scrutinized variables in the models.

### Model comparison

Models were compared using the Bayesian deviance information criterion (DIC) [39] with smaller values (with a difference of at least 7 units) indicating an improvement in model fit [39].

All available covariates (Table 1) were initially used for the multivariate discrete-time multilevel logistic survival analysis. We initially ran a series of MLwiN models based on the likelihood method [37] to determine those variables that were not significant (p > 0.20) and so excluded from the final models. To explore the impact of unknown stage at diagnosis on model fit and summary measures, sensitivity analyses were carried out by repeating the all-cause and CRC-specific survival analyses under three different assumptions; (a) all unstaged cases being reclassified as Stage I, b) reclassified as Stage IV or c) equally distributed over all four stage categories.

Fixed parameter estimates are presented as odds ratios (OR) with their 95% credible intervals (CrI). Joint chi-square tests were used to assess the contribution of each variable to model fit.

### The median odds ratio

The median odds ratio (MOR) [40,41] is a measure of the variation between the mortality rates of different SLAs that is not explained by the modeled risk factors. It is expressed in terms of the odds ratio scale. If the MOR is equal to 1 there is no difference between areas. Larger values indicate greater geographical variation in survival. The MOR was calculated for the discrete-time multilevel logistic survival models as:

$$\text{MOR}=\exp\;\left(Z_{0.75}\times \sqrt{2\sigma^{2}}\right)$$

where Ζ_0.75_ is the 75th percentile of the normal distribution and *σ*^2^ is the estimated area-level variance from the MCMC simulations. A 95% CrI for the MOR was generated from the posterior distribution of the variance [30].

### The interval odds ratio

In multilevel modeling, the interpretation of an area-level risk factor such as remoteness or area disadvantage should be interpreted as the effect of the risk factor given a comparison between two SLAs of identical values of the random effect whose mortality probabilities differ only in terms of the risk factor under consideration [41]. Therefore, to interpret the area-level risk factors more generally, the unexplained between-area variability also needs to be taken into account. This is achieved using the 80% interval Odds Ratio (IOR) [30], which shows the impact of area-level risk factors on mortality when comparing SLAs with different area-level characteristics. The IOR is calculated as:

$$\mathrm{IO}R_{\text{lower}/\text{upper}}=\exp\;\left(\beta +Z_{0.10/0.90}\times \sqrt{2\sigma^{2}}\right)$$

where β is the regression coefficient for the area-level variable, *σ*^2^ is the area-level variance and Z_0.10_ and Z_0.90_ are the 10th and 90th centiles respectively of the standard normal distribution. If the IOR does not include 1.0 it indicates that the effect of the area-level variable is large relative to the clustering effect of the SLAs.

## Results

### Study population

Between 1997 and 2007 there were 25,788 invasive CRC cases in Queensland. Of these 23,634 were aged 20–84 years at diagnosis who initially comprised the study cohort. The exclusion of cases that had incomplete address at diagnosis information (n = 723), were identified at autopsy (n = 33), had death certificate notification only (n = 126) or who survived for less than one day (n = 25) gave the final cohort of 22,727 cases.

Among the final cohort (Table 1), approximately 37% of cancers were diagnosed at advanced stage of which one third (31%) had metastatic (Stage IV) disease. There were 9,337 (41.1%) deaths during the first five years after diagnosis of which 7,221 were attributed to CRC.

### Bivariate Kaplan-Meier survival analysis

**Figure 1:**
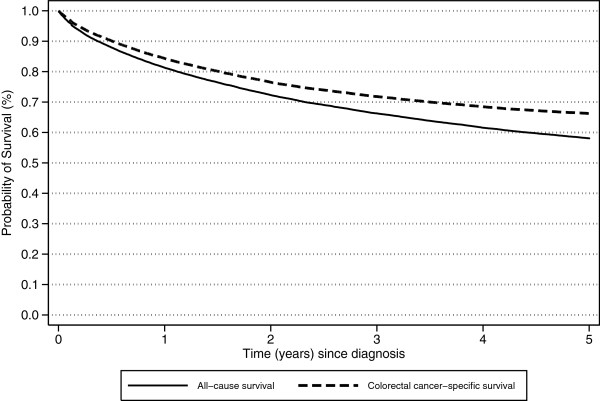
Kaplan-Meir survival curves for the cumulative probability of all-cause and colorectal cancer-specific survival five years from diagnosis for colorectal cancer patients aged 20–84 in Queensland, 1997–2007.

**Figure 2:**
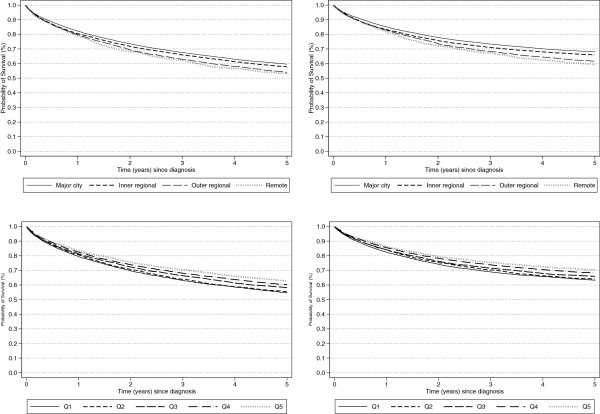
**Kaplan-Meir five-year survival curves (from diagnosis) for colorectal cancer patients aged 20–84 in Queensland, 1997–2007 by geographic remoteness (early: n = 13,155; inner regional: n = 5,139; outer regional: n = 3,485; remote: n = 948) and area socio-economic disadvantage which was categorized into 5 quintiles of increasing advantage from Quintile 1 (Quintile 1: n = 3,023; 2: n = 5,335; 3: n = 6,075; 4: 5,101; 5: 3,193). a)** all-cause survival by remoteness **b)** colorectal cancer-specific survival by remoteness **c)** all-cause survival by area disadvantage **d)** colorectal cancer-specific survival by area disadvantage.

The unadjusted 5-year all-cause and CRC-survival rates were 58.1% (95% CI: 57-58%) and 66.3% (95% CI: 66-67%) respectively (Figure 1). For both survival measures there was a difference of about 6–8 percentage points between people living in the most remote areas and those from major cities, and also between residents of the most and least disadvantaged areas. (Table 1; Figure 2) All-cause and CRC-specific survival decreased with increasing age, longer travel distances, poorer tumor differentiation or higher stage at diagnosis with poorer survival also seen for patients who were Indigenous, blue collar workers, unmarried, males or born in non-English-speaking countries.

### Discrete-time multilevel logistic survival analysis

#### Development of final all-cause survival model

Based on the DIC measure, model fit was markedly improved by adding the individual effects to the null model for all-cause survival (Model 2). Adding in remoteness (Model 3) or area disadvantage (Model 4) further reduced the DIC by at least 7 units. Comparing the DIC statistic of these models with the fully adjusted main-effects model (Model 5) suggested that Model 5 provided an improved fit (Table 2). The additional introduction of the area-level interaction term (Model 6) did not reduce the DIC statistic; hence we retained Model 5 as the final model for all-cause survival. Parameter estimates presented here refer to this model.

**Table 2:** Measures of model fit and estimates of geographical variations in all-cause and colorectal cancer-specific survival in Queensland, 1997–2007

**Model**	**description**^**1**^	**DIC**^**2**^	**Area-variance (95% CrI)**^**3**^	**p value**	**% reduction variance**^**4**^	**MOR (95% CrI)**^**5**^
	*All-cause survival*					
1	Null (no explanatory variables)	57769.71	0.025 (0.014, 0.039)	<0.001	-	1.16 (1.13, 1.21)
2	Individual-level covariates^5^	46072.45	0.011 (0.006, 0.018)	0.042	56	1.10 (1.08, 1.14)
3	Individual-level covariates and area-remoteness	46058.44	0.007 (0.003, 0.014)	0.101	72	1.08 (1.05, 1.12)
4	Individual-level covariates and area-disadvantage	46054.03	0.006 (0.001, 0.014)	0.078	76	1.08 (1.03, 1.12)
5	Individual- and both area-level covariates	46046.57	0.005 (0.001, 0.012)	0.118	80	1.07 (1.03, 1.11)
6	All covariates with area-level interactions	46051.12	0.005 (0.001, 0.012)	0.128	80	1.07 (1.03, 1.11)
	*Colorectal cancer-specific survival*					
7	Null (no explanatory variables)	48609.89	0.021 (0.011, 0.034)	0.001	-	1.15 (1.10, 1.19)
8	Individual -level covariates	37013.72	0.008 (0.003, 0.012)	0.044	61	1.09 (1.05, 1.11)
9	Individual-level covariates and area-remoteness	36987.44	0.003 (0.001, 0.007)	0.231	86	1.05 (1.02, 1.08)
10	Individual-level covariates and area-disadvantage	36995.14	0.004 (0.001, 0.011)	0.173	81	1.06 (1.02, 1.10)
11	Individual- and both area-level covariates	36979.49	0.002 (0.001, 0.008)	0.231	91	1.04 (1.02, 1.09)
12	All covariates with area-level interactions	36993.31	0.003 (0.001, 0.011)	0.298	86	1.05 (1.02, 1.10)

CrI: credible interval for posterior distributions from Markov Chain Monte Carlo multilevel models.

^1^ All except null models adjusted for time (years after diagnosis), patient age at diagnosis, sex, occupation, marital and Indigenous status, cancer stage, site, differentiation and surgical margins. Models 3 and 9 also adjusted for area remoteness; Models 4 and 10 also adjusted for area disadvantage; Models 5 and 11 also adjusted for area remoteness and area disadvantage; Models 6 and 12 include area-level interactions and all explanatory factors in Models 5 or 11 respectively.

^2^ DIC Deviance Information criterion. Model with a smaller DIC (Difference ≥ 7 units) is better supported by the data while those with difference of 3–5 units can be weakly distinguished.

^3^ The residual area-level variance from the multilevel models.

^4^ The percentage reduction in variance reflects the change in area-level variance relative to the null model.

^5^ MOR (median odds ratio) translate the area-level variance to the odds ratio scale. MOR is the median value of the distribution of the odds ratio between an individual in the area at highest risk and an individual in the area at lowest risk when randomly selecting two individuals from two different areas. In absence of variation the MOR is 1 with a larger value indicating greater geographical variations.

#### Development of final CRC-specific survival model

The DIC statistic indicated that adjusting for individual effects (Model 8) significantly improved fit over the null model (Model 7). The DIC was further reduced by at least 7 units on introduction of remoteness (Model 9) or area disadvantage (Model 10). Based on DIC criteria model fit was further improved for the fully adjusted main-effects Model 11 (Table 2) while overall fit of the interaction model (Model 12) was poorer than its main-effects counterpart. Therefore we considered model 11 to be best fitting model for these CRC-survival data and used it for the remainder of this analysis.

#### Area-level interactions

Interactions between geographic remoteness and area disadvantage were also not statistically significant for all-cause (Wald χ^2^ = 12.22, df = 11, p = 0.347) and CRC-specific (Wald χ^2^ = 8.83, df = 11, p = 0.638) survival, implying that the impact of socioeconomic disadvantage on both all-cause and CRC-survival were similar for urban and rural CRC patients.

#### Area-level variance

The null models indicated significant evidence of geographical variation in both all-cause (Model 1; p < 0.001) and CRC-specific (Model 7; p = 0.001) survival across 478 SLAs in Queensland (Table 2). However, when successively adding the individual-level and area-level variables to the models, the amount of unexplained geographical variation decreased, to which point it became non-significant for the final model for both all-cause (Model 5, p = 0.118) and CRC-specific survival (Model 11; p = 0.231). This lack of statistical significance was reflected in the relatively low values (i.e. close to one) of the MORs in Table 2.

#### Impact of area-level covariates on area-level variation

All of the IOR-80 intervals (Table 3) by area-disadvantage were relatively ‘narrow’ and did not contain 1, suggesting that the impact that area-level disadvantage quintiles had on survival was large relative to the clustering effect of the SLAs. The impact of remoteness was less clear; while there was no evidence (IOR interval contained 1) that the difference between major cities and inner regions had an impact on the area-level variation; there was some difference between major city and remote areas.

**Table 3:** Interval odds ratios (80%) for the influence of area disadvantage or remoteness on geographical variations in all-cause and colorectal cancer survival in Queensland, 1997–2007

	**IOR-80**^**1,2**^	
	*All-cause survival*	*Colorectal cancer-specific survival*
	Model 5^3^	Model 11^3^
**Area-Remoteness Index of Australia (ARIA)**		
*Major city*	*1.00*	*1.00*
*Inner regional*	0.89, 1.15	0.90, 1.06
*Outer regional*	0.96, 1.24	1.06, 1.25
*Remote*^*4*^	1.01, 1.31	1.14, 1.34
**Index of Relative socioeconomic advantage and disadvantage (IRSAD)**		
*Quintile 5 (least disadvantaged)*	*1.00*	*1.00*
*Quintile 4*	1.01, 1.30	1.03, 1.21
*Quintile 3*	1.04, 1.34	1.06, 1.25
*Quintile 2*	1.07, 1.39	1.11, 1.30
*Quintile 1 (most disadvantaged)*	1.10, 1.42	1.13, 1.39

^1^ IOR-80 is the 80% interval odds ratio which quantifies the contribution of area-level explanatory factors to the variations in survival between areas. If the interval does not include 1.0 it indicates that the effect of the area-level variable is large relative to the clustering effect of the SLAs.

^2^ IOR-80 is calculated with respect to the reference category for each area-level explanatory variable.

^3^ Estimates derived from best fitting fully adjusted Models 5 and 11 respectively (see text for details).

^4^ Includes remote and very remote categories.

#### Fixed parameter estimate

Independent of individual-level factors, both area disadvantage (p = 0.004) and geographic remoteness (p < 0.001) were significantly associated with CRC-cancer specific survival (Table 4). Statistically significant associations were also evident between area disadvantage (p < 0.001) and remoteness (p =0.003) with all-cause survival.

**Table 4:** Geographic remoteness, area-disadvantage and the adjusted odds of all-cause and colorectal cancer mortality in Queensland, 1997–2007

	**All-cause**	***p***	**Colorectal cancer-specific**	***p***
sub group	OR (95% CrI)^1^		OR (95% CrI)^1^	
***Area-Remoteness Index of Australia (ARIA)***		*=0.004*		*=0.003*
Major city	1.00		1.00	
Inner regional	0.95 (0.88, 1.02)		0.98 (0.90, 1.06)	
Outer regional	1.09 (1.01, 1.18)		1.15 (1.05, 1.25)	
Remote^2^	1.15 (1.02, 1.28)		1.24 (1.07, 1.42)	
p-value				
***Relative socioeconomic advantage and disadvantage (IRSAD)***		*<0.001*		*<0.001*
Quintile 5 (least disadvantaged)	1.00		1.00	
Quintile 4	1.14 (1.03, 1.23)		1.12 (1.01, 1.23)	
Quintile 3	1.18 (1.08, 1.29)		1.15 (1.03, 1.27)	
Quintile 2	1.22 (1.11, 1.34)		1.20 (1.07, 1.33)	
Quintile 1 (most disadvantaged)	1.25 (1.10, 1.36)		1.23 (1.09, 1.30)	
***Time (years after diagnosis)***	0.45 (0.41, 0.50)	*<0.001*	0.47 (0.42, 0.52)	*<0.001*
***Time-squared ([years after diagnosis]squared])***	1.07 (1.05, 1.08)	*<0.001*	1.04 (1.02, 1.07)	*<0.001*
***Age group***		*<0.001*		*<0.001*
20 to 49	0.24 (0.21, 0.27)		0.37 (0.33, 0.43)	
50 to 59	0.29 (0.26, 0.32)		0.45 (0.41, 0.51)	
60 to 69	0.42 (0.39, 0.46)		0.59 (0.54, 0.65)	
70 to79	0.68 (0.63, 0.73)		0.77 (0.70, 0.84)	
80 to 85	1.00		1.00	
***Gender***		*<0.001*		*<0.001*
Male	1.00		1.00	
Female	1.08 (1.02, 1.14)		1.17 (1.10, 1.25)	
***Marital status***		*<0.001*		*<0.001*
Married	1.00		1.00	
Never married/ single	1.33 (1.21, 1.46)		1.29 (1.16, 1.43)	
Widowed	1.11 (1.03, 1.19)		1.06 (0.97, 1.14)	
Divorced	1.18 (1.08, 1.29)		1.17 (1.06, 1.29)	
Separated	0.94 (0.77, 1.13)		0.89 (0.71, 1.09)	
Not stated	1.32 (1.02, 1.68)		1.41 (1.03, 1.85)	
***Occupation category***		*<0.001*		*<0.001*
Professional	1.00		1.00	
White collar	1.11 (1.02, 1.20)		1.07 (0.97, 1.17)	
Blue collar	1.38 (1.29, 1.49)		1.36 (1.25, 1.47)	
Not in labor force	0.46 (0.43, 0.50)		0.50 (0.46, 0.54)	
Not stated/ Inadequately described	0.35 (0.32, 0.39)		0.34 (0.31, 0.38)	
***Country birth***^***3***^		*=0.045*		*=0.040*
Australia	1.00		1.00	
Other english-speaking	0.96 (0.90, 1.02)		0.99 (0.92, 1.06)	
Non-english-speaking	0.88 (0.76, 0.97)		0.87 (0.74, 0.86)	
***Indigenous status***		*<0.001*		*<0.001*
Non indigenous	1.00		1.00	
Indigenous	1.16 (0.89, 1.49)		1.09 (0.99, 1.44)	
Not stated	0.45 (0.39, 0.51)		0.43 (0.36, 0.50)	
***Site***^***4***^		*=0.076*		*=0.134*
Proximal (R) colon	1.02 (1.01, 1.08)		1.01 (0.99, 1.13)	
Distal (L) colon	1.03 (0.96, 1.10)		0.97 (0.93, 1.00)	
Colon NOS	1.04 (1.01, 1.16)		1.02 (0.99, 1.07)	
Rectal	1.00		1.00	
***Stage***		*<0.001*		*<0.001*
Stage A	1.00		1.00	
Stage B	1.61 (1.47, 1.77)		2.44 (2.15, 2.77)	
Stage C	3.17 (2.91, 3.45)		6.09 (5.40, 6.85)	
Stage D	11.41 (10.30, 12.57)		23.25 (20.39, 26.43)	
Unknown stage	2.09 (1.86, 2.34)		3.59 (3.09, 4.14)	
***Differentiation***		*<0.001*		*<0.001*
Well differentiated	1.00		1.00	
Moderate differentiated	1.14 (1.01, 1.29)		1.37 (1.18, 1.60)	
Poor differentiated	1.64 (1.44, 1.87)		2.07 (1.77, 2.43)	
Not stated differentiation	1.25 (1.09, 1.43)		1.46 (1.24, 1.73)	
***Surgical margins***		*<0.001*		*<0.001*
Clear	1.00		1.00	
Positive	1.42 (1.19, 1.66)		1.55 (1.27, 1.85)	
Unknown margin	1.84 (1.68, 2.01)		1.97 (1.79, 2.17)	

CrI: credible interval for posterior distributions from Markov Chain Monte Carlo multilevel models.

^1^ Estimates derived from best fitting fully adjusted Models 5 and 11 (see text for details).

^2^ Includes remote and very remote categories.

^3^ Other English-speaking: those born in New Zealand, United Kingdom, Ireland, or North America; non-English-speaking: those not born in Australia, New Zealand, United Kingdom, Ireland or North America.

^4^ Colorectal sites defined as: proximal colon (ICDO3: C180 to C184), distal colon (ICDO3: C185-C187), unspecified colon (ICDO3: C188-C189) and rectal (ICDO3: C19-C20, C218).

Compared to CRC patients from the least disadvantaged quintile (Quintile 5), residents of the remaining four quintiles had worse CRC-specific (OR 1.12, 1.15, 1.20, 1.23 for Quintiles 4, 3, 2 and 1 respectively) and all-cause survival (OR ranging from 1.14 to 1.25 by quintiles of increasing disadvantage). Compared to those from major cities, living in outer regional and remote areas was also associated with significantly poorer all-cause (outer regional: OR 1.09, remote: OR 1.15) and CRC-specific survival (outer regional: OR 1.15, remote: OR 1.24).

In addition to increasing age and stage, all other individual-level clinical and socio-demographic factors (except site were independent predictors of both all-cause and CRC-specific survival in multivariate analysis. (Table 4) Finally CRC survival decreased with time (with a quadratic relationship between survival and years of follow-up). There was little difference in the parameter estimates for all the fixed effects across each set of models (full results not shown).

The sensitivity analyses for stage (full results not shown) suggested that the independent association of area-level remoteness and disadvantage with both all-cause and CRC-specific survival remained regardless of the proposed assumptions for the true distribution of cancer stage at diagnosis.

## Discussion

In this large population-based study of CRC patients in Queensland we found that survival outcomes depended on where patients lived at diagnosis, and that this disparity remains after adjustment for important individual-level socio-demographic and clinical factors. Specifically our results demonstrated that residents of more disadvantaged and remote areas had significantly lower all-cause and CRC-specific survival five years after diagnosis of CRC, irrespective of their individual characteristics and irrespective of the clinical characteristics of their cancers, including cancer stage at diagnosis.

There are a number of potential explanations for the observed survival disparity including possible differences in management patterns, although without more information these remain speculative. Geographical barriers and poorer health infrastructure [12] have previously been associated with lower receipt of multimodal therapies and lower survival of CRC patients in rural or (outer) regional Australia compared to major cities, [8,42,43] and, internationally, the impact on patient outcomes of variations in hospital volume and clinical experience are well-documented [44-46] in that higher caseloads and increased specialization generally improve CRC-related outcomes. All major centers of oncological care in Queensland are located in metropolitan areas and this is likely to be part of the explanation for the relatively better outcomes in major city and inner regional areas.

However we also found strong evidence of lower survival outcomes for people living in socioeconomically disadvantaged areas, irrespective of their remoteness and other individual and clinical characteristics, meaning that distance is not the only explanation. While Australia does have universal free hospital cover, previous research has shown that colorectal cancer patients who seek medical care in private hospitals have experienced better outcomes [10]. It is also possible that these area-level effects may at least partially reflect geographical differences in the distribution of other important patient characteristics that are known to influence prognosis, such as overweight, physical inactivity, smoking, dietary patterns, comorbidities and general health status as well as treatment [4-6,9,42]. For example people living in socioeconomically disadvantaged or rural areas are more likely to engage in high risk behaviors such as smoking and decreased physical activity [47]. In addition, people living in rural and remote areas of Australia have a higher prevalence of comorbidities such as diabetes and cardiovascular conditions that can significantly impact the clinical management and prognosis for CRC patients [48]. The impact of selected individual risk factors on geographical differences in all-cause mortality for Australia has been recently quantified and suggests that interventions targeted at modifiable health factors could translate to a substantial reduction (around 36-45%) in the regional mortality differentials [49].

Since the vast majority of people diagnosed in Queensland over the current study period (1996–2007) were symptomatic [50] and the gradual implementation of the National Bowel Cancer Screening Program [51] only began in late 2006; any influence that screening had on current results would be limited. However geographical differences in participation rates may impact CRC outcomes in the future.

Important strengths of this study include the population-based coverage and high quality of incidence data from the Queensland cancer registry [17] and inclusion of all routinely available covariates in the analysis. Disease stage was clinically coded from pathological forms. All-cause survival was estimated, along with CRC-specific survival, thereby avoiding the intrinsic dependence of cause-specific survival on cause-of death coding [52]. The multilevel design takes into account the hierarchical data structure and allows the simultaneous estimation of both individual- and area-level effects on survival, something that is not possible in ecological studies. The estimated random effects from MCMC simulations were quantified using MOR [30,53] to assess the magnitude of geographic variation in a meaningful way. In addition the IOR-80 interval which integrates area-level fixed and random terms was used to quantify area-level covariate effects in comparison to the unexplained variation [30].

However the Queensland cancer registry does not collect information on potential confounders including but not limited to treatment, life style, comorbidities, family history, ongoing surveillance, stress, inflammation and other measures of individual socioeconomic status (income and education) [4,5,54-56]. Different measures of socioeconomic status are not interchangeable and can have a diverse impact on health outcomes [57]. The occupation measure used for the current study was limited in its sensitivity and precision since it was not possible to disaggregate the ‘Not stated/Inadequately described category into more homogenous groupings such as ‘home duties’, ‘retired’ or unemployed’ based on available information. Around 16% of cases could not be staged and are likely to be fairly advanced at diagnosis (based on 5-year survival estimates by stage) however sensitivity analyses confirmed that the results were consistent under the various assumptions of the missing stage information.

## Conclusions

This study has demonstrated that people diagnosed with CRC in rural and disadvantaged areas have significantly poorer survival than those living in urban and affluent areas, independently of cancer stage and other individual-level characteristics. Addressing this survival disadvantage will require as a first step a commitment of resources to clarify and quantify the main causes for this disparity, and it is hoped that these results provide the necessary motivation and impetus for this to happen. The causes of these inequalities are likely to be complex and difficult to unravel, however, a better understanding is essential to inform the development of interventions to improve survival in rural and disadvantaged areas to the level of the rest of the population.

## Abbreviations

CRC: Colorectal cancer; IRSAD: Index of relative socioeconomic advantage and disadvantage; SLA: Statistical local area; MCMC: Markov chain Monte Carlo; MOR: Median odds ratio; CrI: Credible interval; DIC: Deviance information criterion; OR: Odds ratio; IOR: Interval odds ratio.

## Competing interests

The authors declare they have no competing interests.

## Authors’ contributions

PB, GT and JA conceived the study, PD conducted the data analysis, PB and PD drafted the manuscript and PD, PD, JA and GT refined and approved the final version of the paper. All authors read and approved the final manuscript.

## Pre-publication history

The pre-publication history for this paper can be accessed here:

http://www.biomedcentral.com/1471-2407/13/493/prepub

## Supplementary Material

Additional file 1 Parameter estimates from Cox and logistic regression models for colorectal cancer patients aged 20–84 in Queensland, 1997–2007. Click here for file

Additional file 2 Comparison of Parameter estimates from Cox model and a logistic regression model. Click here for file
