# Supplementary material for: Geographic remoteness, area-level socioeconomic disadvantage and inequalities in colorectal cancer survival in Queensland: a multilevel analysis
Source: BMC Cancer. 2013 Oct 24;13:493. doi: 10.1186/1471-2407-13-493 (PMC3871027; doi:10.1186/1471-2407-13-493)
Supplement: Additional file 2 — Comparison of Parameter estimates from Cox model and a logistic regression model. [file 1471-2407-13-493-S2.pdf]

Comparison of Parameter estimates from Cox model and a logistic regression model

In Additional Table 1 the parameter estimates from a standard Cox regression model on the original dataset, and the logistic regression model on the expanded person-period dataset are compared. These models were only fitted to a limited number of covariates and ignore the multilevel structure of the data. However they illustrate that censoring is treated the same way with both analytical approaches and that logistic regression with a person-period dataset is a good approximation to Cox modeling.
